# Granzyme B PET Imaging Reveals Lgmn^+^ Macrophage‐Mediated Immune Evasion and Guides Immunotherapy in EGFR‐TKI‐Resistant NSCLC

**DOI:** 10.1002/advs.77837

**Published:** 2026-09-16

**Authors:** Dongliang Wang, Wenqin Luo, Qiufang Liu, Wenxuan Tang, Bin Zhu, Jiwei Chen, Qian Zhou, Shuang Tang, Xiaoping Xu, Shaoli Song

**Affiliations:** ^1^ Department of Nuclear Medicine Shanghai Cancer Center Fudan University Shanghai China; ^2^ Department of Oncology Shanghai Medical College Fudan University Shanghai China; ^3^ Shanghai Engineering Research Center of Molecular Imaging Probes Shanghai China; ^4^ Center For Biomedical Imaging Fudan University Shanghai China; ^5^ Key Laboratory of Nuclear Physics and Ion‐beam Application (MOE) Fudan University Shanghai China; ^6^ Shanghai Key Laboratory of Molecular Imaging Shanghai University of Medicine and Health Sciences Shanghai China; ^7^ Department of Colorectal Surgery Fudan University Shanghai Cancer Center Shanghai China; ^8^ Department of Digestive Diseases and National Clinical Research Center for Aging and Medicine Huashan Hospital Fudan University Shanghai China

**Keywords:** ^68^Ga‐GZB PET/CT imaging, EGFR‐TKI, extracellular vesicles, immunotherapy, Lgmn^+^ macrophages, non‐small cell lung cancer

## Abstract

Determining optimal therapies for EGFR‐mutant NSCLC after tyrosine kinase inhibitor (TKI) resistance remains challenging. Immune checkpoint inhibitors (ICIs) show limited efficacy, but some patients regain immunotherapy sensitivity post‐TKI. Non‐invasive monitoring to identify responders and understand persistent immune tolerance is needed. In this study, we prospectively evaluated a novel granzyme B‐targeted PET radiotracer, ^68^Ga‐GZB, in a clinical cohort of NSCLC patients. ^68^Ga‐GZB PET/CT accurately visualized early immune responses and stratified patients likely to benefit from immunotherapy. In preclinical models of acquired EGFR‐TKI resistance, we tracked dynamic immune reactivity via ^68^Ga‐GZB PET/CT. Single‐cell transcriptomics and functional validation identified Lgmn^+^ macrophages as a highly immunosuppressive subset driving persistent immunotherapy tolerance. Mechanistically, Lgmn^+^ macrophages secrete extracellular vesicles (EVs) that deliver Lgmn directly into CD8^+^ T cells. Internalized Lgmn recruits the E3 ubiquitin ligase Chip, initiating the ubiquitination and proteasomal degradation of the glycolytic enzyme Eno1. This cascade severely impairs CD8^+^ T cell glycolysis, ultimately leading to CD8^+^ T cell exhaustion. Furthermore, we demonstrate that targeting of Lgmn synergizes with PD‐1 blockade to reactivate anti‐tumor immunity in TKI‐resistant models. Thus, ^68^Ga‐GZB PET/CT provides a non‐invasive platform to stratify immunotherapy responses, and targeting Lgmn represents a novel combination strategy for EGFR‐TKI‐resistant NSCLC.

## Graphical abstract

Graphical illustration of ^68^Ga‐GZB PET/CT imaging reveals immune dynamics post‐EGFR‐TKI resistance in NSCLC; Lgmn^+^ macrophages‐derived Lgmn‐containing extracellular vesicles inhibit glycolysis, induce CD8^+^ T cell exhaustion, and targeting Lgmn restores immunotherapy sensitivity.

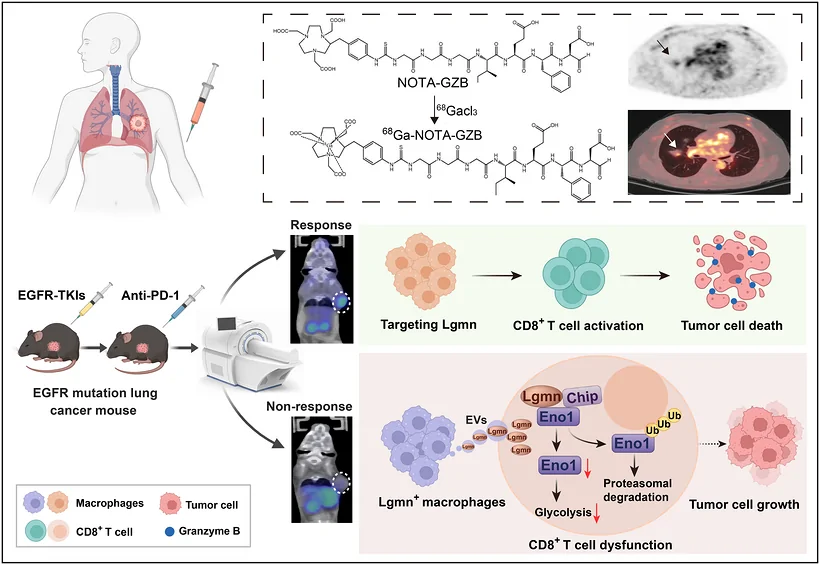

## 1 Introduction

Immune checkpoint inhibitors (ICIs) have fundamentally transformed the therapeutic landscape for non‐small cell lung cancer (NSCLC) [1]. However, for patients with EGFR mutations, especially those who have reached a therapeutic impasse due to acquired resistance to TKI, whether immunotherapy can serve as a viable strategy remains a central clinical concern. Current evidence indicates that TKI therapy can remodel the tumor immune microenvironment (TIME) in EGFR‐mutant lung cancer, providing a vital theoretical rationale for the application of immunotherapy in this population [2, 3]. Nevertheless, clinical practice reveals that only a select subset of patients truly derive benefit. Hence, precise stratification of potential responders, implementation of dynamic immune monitoring, and elucidation of the mechanisms sustaining immune evasion have become critical to overcoming the current treatment bottleneck.

Positron emission tomography/computed tomography (PET/CT), as a non‐invasive whole‐body imaging modality, is recognized as the optimal approach for early evaluation of tumor efficacy [4]. Although ^18^F‐fluorodeoxyglucose (^18^F‐FDG), the most commonly used PET tracer, is widely applied to assess tumor glucose metabolism, it lacks the specificity to capture early immune activation accurately [4]. Recently, novel molecular probes targeting CD8^+^ T cells or the PD‐L1/PD‐1 axis have entered clinical evaluation [5, 6], however, these tracers primarily quantify immune cell presence or target abundance, which may not necessarily reflect the functional and cytotoxic status of the anti‐tumor immune response. Given that effective T cell‐mediated cytotoxicity ultimately depends on the release of perforin and granzyme B [7], visualizing granzyme B secretion provides a direct and functional readout of active anti‐tumor immunity. Building on our previous development of ^68^Ga‐GZB, a highly specific PET radiotracer targeting granzyme B [8], we initiated a prospective clinical study to validate its predictive value in NSCLC patients. We hypothesized that ^68^Ga‐GZB PET/CT could serve as a powerful, non‐invasive imaging biomarker to monitor early immune reactivity and accurately stratify responders from non‐responders in clinical practice.

In addition to clinical monitoring, there is an equally urgent need to elucidate the mechanisms driving persistent immune evasion in EGFR‐mutant lung cancer. The TIME is a highly orchestrated cellular and molecular network characterized by complex regulation of anti‐tumor immunity [9]. Although CD8^+^ T cells and natural killer (NK) cells are the primary effectors of tumor elimination, their activity is often suppressed by an immunosuppressive niche sustained by macrophages, myeloid‐derived suppressor cells (MDSCs), and cancer‐associated fibroblasts (CAFs) [10]. This antagonistic crosstalk markedly affects immunotherapeutic efficacy. Consequently, elucidating the sophisticated intercellular crosstalk within this network is imperative not only for understanding TIME plasticity but also for uncovering novel therapeutic vulnerabilities that could enhance the efficacy of immunotherapy in EGFR‐TKI‐resistant NSCLC. To dissect this intricate crosstalk, we applied the clinically validated ^68^Ga‐GZB PET/CT imaging to preclinical models of EGFR‐mutant lung cancer, successfully capturing the dynamic evolution of immunoreactivity following the acquisition of EGFR‐TKI resistance.

Using this imaging‐guided stratification alongside single‐cell RNA sequencing (scRNA‐seq), we identified a highly immunosuppressive subset of Lgmn^+^ macrophages that accumulate in TKI‐resistant tumors. Mechanistically, we uncovered a novel mode of intercellular communication: Lgmn^+^ macrophages secrete extracellular vesicles (EVs) that deliver the Lgmn protein directly into CD8^+^ T cells. Once internalized, Lgmn recruits the E3 ubiquitin ligase Chip to promote the ubiquitination and degradation of the glycolytic enzyme Eno1, thereby impairing CD8^+^ T cell glycolysis and driving functional exhaustion. Crucially, we show that targeting Lgmn alleviates this metabolic suppression, significantly enhancing the efficacy of PD‐1 blockade in TKI‐resistant models. Collectively, this study establishes ^68^Ga‐GZB PET/CT as a robust clinically translatable tool for dynamic immune monitoring, enabling the precise identification of EGFR‐mutant patients likely to benefit from immunotherapy after TKI resistance. Furthermore, we identify Lgmn‐targeted combination therapy as a highly promising strategy to overcome the immunotherapy bottleneck in NSCLC after TKI failure.

## 2 Results

### 2.1 ^68^Ga‐GZB PET/CT Serves as a Robust Non‐Invasive Biomarker for Early Immunotherapy Response in a Prospective NSCLC Cohort

**FIGURE 1:**
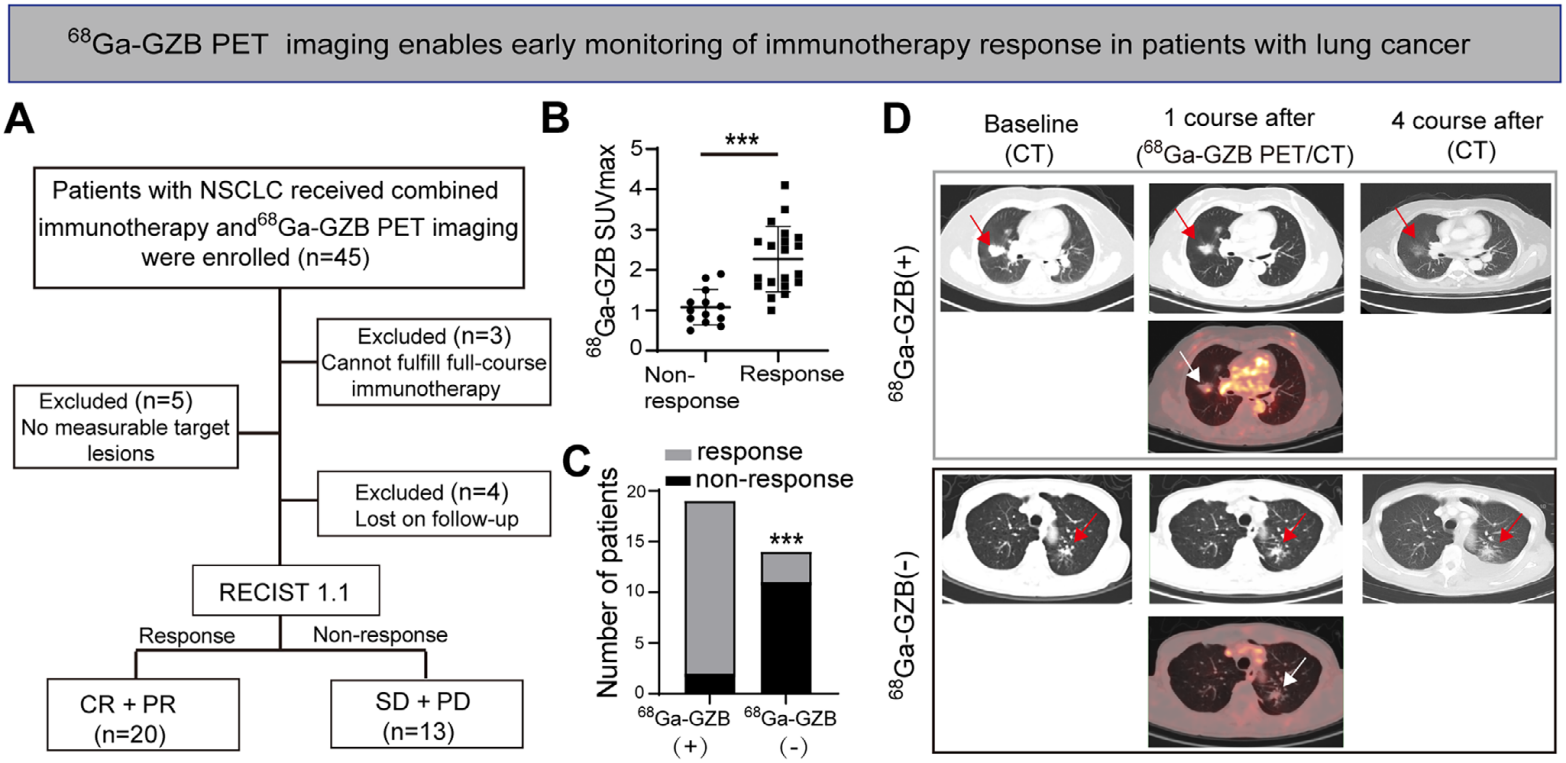
^68^Ga‐GZB PET/CT effectively assesses early immunotherapy response in NSCLC patients. (A) Flow chart of the clinical research technology route. (B) ^68^Ga‐GZB uptake values of NSCLC patients in the immunotherapy‐responsive and non‐responsive groups. (C) Percentage of immunotherapy response in ^68^Ga‐GZB (+) and ^68^Ga‐GZB (‐) NSCLC patients. (D) The chemical structural formula of the ^68^Ga‐GZB probe is shown at the top of the figure, and representative images of ^68^Ga‐GZB (+) and ^68^Ga‐GZB (‐) NSCLC patients are shown at the bottom of the figure, respectively.

To establish the clinical utility of our previously developed granzyme B‐targeting PET radiotracer, ^68^Ga‐GZB (^68^Ga‐NOTA‐GGGG‐IEPD‐CHO) (Figure S1A), we conducted a prospective clinical imaging study to monitor early immunotherapy responses in patients with advanced NSCLC. We initially enrolled 45 NSCLC patients who underwent anti‐PD‐1/PD‐L1 blockade therapy, and ^68^Ga‐GZB PET/CT imaging was enrolled. After applying predefined exclusion criteria (Figure 1A), 33 eligible patients were retained for subsequent efficacy evaluation (Table S1). According to RECIST 1.1 criteria, the cohort comprised 20 responders (complete or partial response, CR/PR) and 13 non‐responders (stable or progressive disease, SD/PD).

Quantitative analysis of primary tumors revealed that the maximum standardized uptake value (SUVmax) was significantly higher in responders than in non‐responders (Figure 1B). To evaluate the predictive accuracy of ^68^Ga‐GZB PET/CT, we performed receiver operating characteristic (ROC) curve analysis. The SUVmax exhibited excellent discriminatory power in distinguishing responders from non‐responders, with an area under the curve (AUC) of 0.908 and an optimal SUVmax cutoff of 1.55 (Figure S1B). Using this threshold, we stratified the cohort into ^68^Ga‐GZB‐positive (SUVmax > 1.55) and ^68^Ga‐GZB‐negative (SUVmax ≤ 1.55) groups. Strikingly, patients in the ^68^Ga‐GZB(+) group had an outstanding clinical response rate of 89.5% (17/19), whereas only 21.4% (3/14) of the ^68^Ga‐GZB(‐) group derived clinical benefit (Figure 1C). A ^68^Ga‐GZB(+) patient showed profound tumor regression following a full course of immunotherapy, while a ^68^Ga‐GZB(‐) patient exhibited marked disease progression (Figure 1D). Collectively, these clinical data firmly establish ^68^Ga‐GZB PET/CT as a highly sensitive, non‐invasive platform for early prediction of ICI efficacy, facilitating the precise identification of NSCLC patients likely to benefit from immunotherapy.

### 2.2 ^68^Ga‐GZB PET/CT Monitors Early Responses to ICIs and Distinguishes Heterogeneous Immune Responses After TKI Resistance in EGFR‐Mutant Lung Cancer

**FIGURE 2:**
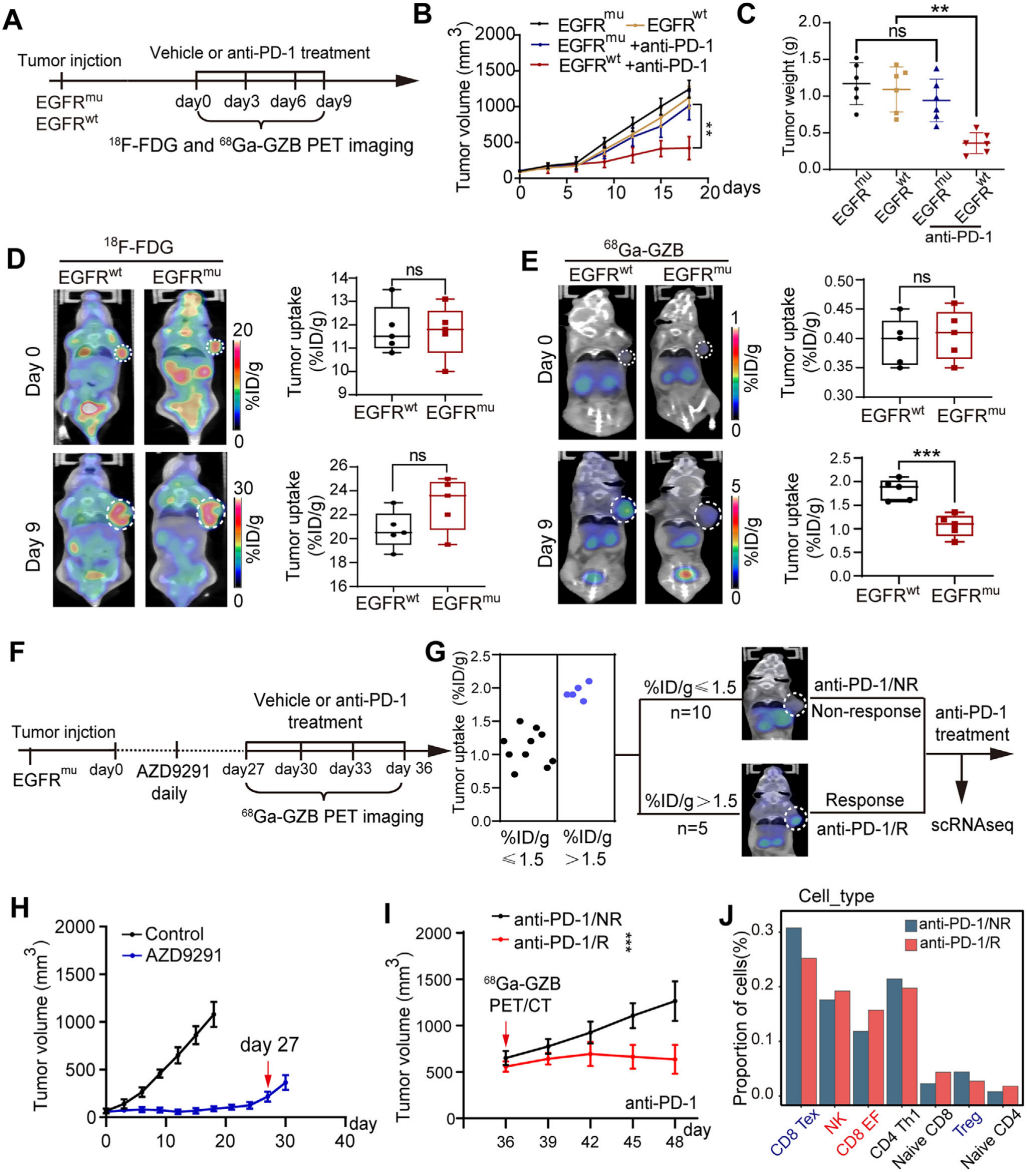
^68^Ga‐GZB PET/CT effectively differentiates different immune responses in EGFR‐TKI‐resistant lung cancer models. (A) Schematic of the anti‐PD‐1 therapy for EGFR wild‐type and EGFR mutant lung cancer mice with ^18^F‐FDG and ^68^Ga‐GZB PET imaging. (B, C) Tumor growth (B) and tumor weight (C) of EGFR wild‐type or EGFR mutant lung cancer treated with control or anti‐PD‐1 mAb (n = 6/group). (D, E) ^18^F‐FDG (D) and ^68^Ga‐GZB (E) PET/CT of EGFR wild‐type or EGFR mutant lung cancer mice treated with control or anti‐PD‐1 mAb at day 0 (upper) and day 9 (lower) (n = 5/group), with representative images on the left and statistical results on the right. (F, G) Schematic of ^68^Ga‐GZB PET/CT differentiating different immune responses in a lung cancer model after resistance to EGFR‐TKI. Firstly, EGFR‐mutant lung cancer mice (n = 15) were treated with AZD9291 daily until tumor volume growth (day 27), and mice were treated with anti‐PD‐1 on day 27 (F). Secondly, ^68^Ga‐GZB PET/CT of mice was performed on day 27 and day 36. With a cut‐off value of 1.5%ID/g (the calculated median uptake), mice were divided into two groups, anti‐PD‐1/NR and anti‐PD‐1/R. Thirdly, scRNA‐seq was performed on the anti‐PD‐1/NR and anti‐PD‐1/R tumors, and the anti‐PD‐1/NR and anti‐PD‐1/R groups of mice were subsequently continued on anti‐PD‐1 treatment (G). (H) Tumor growth of EGFR‐mutant lung cancer mice treated with AZD9291.(I) Tumor growth of anti‐PD‐1/NR and anti‐PD‐1/R mice treated with anti‐PD‐1. (J) Proportion analysis of 7 populations of cell populations.

Having established the broad predictive utility of ^68^Ga‐GZB PET/CT in an NSCLC clinical cohort, we next employed this technology to investigate a formidable clinical challenge: immunotherapy resistance driven by oncogenic EGFR mutations. We established syngeneic murine models using an EGFR‐ex19del mutant Lewis lung carcinoma (LLC) cell line alongside a wild‐type counterpart. Mice were treated with anti‐PD‐1 therapy and underwent concurrent ^18^F‐FDG and ^68^Ga‐GZB PET imaging on days 0 and 9 (Figure 2A). As expected from clinical observations, wild‐type tumors exhibited growth suppression, whereas EGFR‐mutant tumors remained refractory to PD‐1 blockade (Figure 2B,C). While ^18^F‐FDG uptake showed no differences between groups at either time point (Figure 2D), ^68^Ga‐GZB uptake was significantly elevated in wild‐type tumors by day 9 (Figure 2E), which directly correlated with enhanced granzyme B secretion (Figure S2A). Importantly, ^68^Ga‐GZB imaging detected this differential immune activation (day 9) before macroscopic differences in tumor volume emerged (after day 15) (Figure 2B), highlighting its superior sensitivity for early immune monitoring.

To model the acquired TKI resistance and its impact on the tumor immune microenvironment, we treated EGFR‐mutant models with the third‐generation TKI osimertinib (AZD9291), which initially induced tumor growth retardation (Figure 2F–H). Initial tumor regression was inevitably followed by acquired resistance and disease relapse (defined as day 27) (Figure 2H). At the onset of resistance, we initiated sequential anti‐PD‐1 therapy and performed ^68^Ga‐GZB PET imaging on day 36 (Figure 2F). Intriguingly, the TKI‐resistant tumors exhibited heterogeneous spatial uptake of ^68^Ga‐GZB (Figure 2G). Using the established 1.5%ID/g threshold, we stratified the mice into an imaging‐predicted responder group (anti‐PD‐1/R, >1.5%ID/g) and a non‐responder group (anti‐PD‐1/NR, ≤1.5%ID/g) (Figure 2G). Remarkably, subsequent monitoring revealed that anti‐PD‐1 treatment significantly stalled tumor growth in the anti‐PD‐1/R group but yielded minimal efficacy in the anti‐PD‐1/NR group (Figure 2I).

To decode the tumor immune microenvironment alterations governing these divergent responses, we performed single‐cell RNA sequencing (scRNA‐seq) on tumors from both the anti‐PD‐1/R and anti‐PD‐1/NR groups. Cells were annotated into five major types, namely epithelial cells, myeloid cells, T cells, fibroblasts, and endothelial cells, based on established marker gene expression patterns (Figure S2B,C). Quantitative analysis revealed that beyond epithelial cells, T cells and myeloid cells constituted the most abundant immune infiltrates in both groups, with minimal fibroblast and endothelial cell presence (Figure S2D). Subcluster characterization of the T cell compartment revealed that responsive (anti‐PD‐1/R) tumors harbored robust infiltration of cytotoxic CD8^+^ effector T cells (CD8 EF). In contrast, the resistant (anti‐PD‐1/NR) tumors were dominated by exhausted CD8^+^ T cells (CD8 Tex) and regulatory T cells (Tregs) (Figure 2J and Figure S2E,F). These data demonstrate that prior TKI exposure dynamically shapes subsequent ICIs responsiveness and confirms that ^68^Ga‐GZB PET can effectively distinguish heterogeneous immune responses in EGFR‐mutant lung cancer after TKI resistance.

### 2.3 Immunosuppressive Lgmn^+^ Macrophages Accumulate in TKI‐Resistant Tumors and Inversely Correlate With ^68^Ga‐GZB Uptake

**FIGURE 3:**
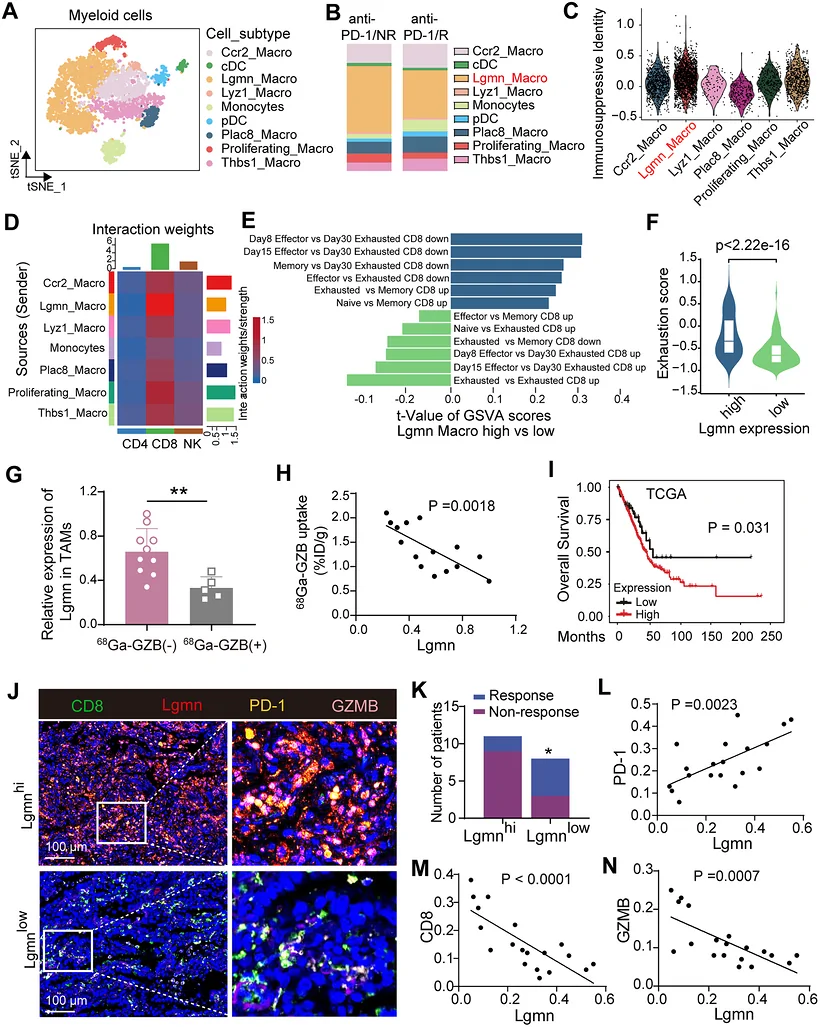
Immunosuppressive Lgmn^+^ macrophage populations are major constituents of the immune‐tolerant EGFR‐TKI‐resistant lung cancer microenvironment. (A) UMAP plot demonstrating the types of myeloid cell populations. (B) Proportion analysis of 9 populations of myeloid cell populations. (C) Immunosuppression‐related gene set scores for macrophage populations. (D) Heatmap of cell‐cell communications in scRNA‐seq analyzed by the CellChat R package. (E) Limma differential analysis of CD8^+^ T cell‐related scores of the GSEA database in Lgmn^+^ macrophage high and low groups. (F) Violin plot of T cell exhaustion score in Lgmn‐high and ‐low groups. (G) *Lgmn* mRNA levels in TAMs from the ^6^ ^8^Ga‐GZB(+) and ^6^ ^8^Ga‐GZB(‐) groups. (H) Correlation analysis of Lgmn with ^6^ ^8^Ga‐GZB uptake. (I) Overall survival analysis of Lgmn in the TCGA database. (J) Multiplex immunofluorescence staining of human lung cancer tissue (n = 19). (K) The proportion of lung cancer patients with and without response to immunotherapy with high and low Lgmn expression. (L–N) Correlation analysis of Lgmn with PD‐1 (L), CD8 (M), and GZMB (N), respectively.

Building on the observation that myeloid cells (aside from epithelial cells) were the most abundant infiltrating population, along with the distinct T cell landscapes revealed by scRNA‐seq, we sought to identify the upstream myeloid populations orchestrating this immune exhaustion. Single‐cell profiling revealed nine distinct myeloid subpopulations with markedly altered distribution patterns between the anti‐PD‐1 non‐responder and responder groups (Figure 3A and Figure S3A). Strikingly, Lgmn‐expressing macrophages (Lgmn^+^ macrophages) emerged as the predominant subset driving this disparity, showing significantly enriched infiltration in anti‐PD‐1/NR tumors (Figure 3B). Functional characterization confirmed that these Lgmn^+^ macrophages possessed the most robust immunosuppressive transcriptomic signature among all myeloid subsets (Figure 3C and Figure S3B). Furthermore, CellChat network analysis identified Lgmn^+^ macrophages as the primary source of immunosuppressive signaling targeting CD8^+^ T cells (Figure 3D). To investigate how Lgmn^+^ macrophages influence CD8^+^ T cell function, we assessed the relationship between Lgmn^+^ macrophages and CD8^+^ T cell status. We observed that high infiltration of Lgmn^+^ macrophages was associated with CD8^+^ T cell exhaustion, whereas low infiltration correlated with effector CD8^+^ T cells (Figure 3E). Furthermore, analysis of public databases revealed that Lgmn expression positively correlated with CD8^+^ T cell exhaustion scores in lung adenocarcinoma (LUAD) (Figure 3F). Intriguingly, macrophages isolated from tumors with high ^68^Ga‐GZB uptake exhibited significantly lower *Lgmn* mRNA levels than those from low‐uptake tumors (Figure 3G). Correlation analysis confirmed a strong negative association between Lgmn expression and ^68^Ga‐GZB uptake (Figure 3H), suggesting that ^68^Ga‐GZB PET imaging can non‐invasively screen for tumors with high Lgmn^+^ macrophage infiltration.

Clinically, using publicly available TCGA data, we found that elevated Lgmn expression was significantly associated with reduced overall survival and poorer prognosis (Figure 3I). Furthermore, analysis of the GSE161537 dataset showed that NSCLC patients with high Lgmn expression in macrophages (Macro‐Lgmn^hi^) exhibited a significantly lower response rate to ICI therapy compared to those with low Lgmn expression (Macro‐Lgmn^low^) (Figure S3C). Moreover, multiplex immunohistochemical (mIHC) analysis of NSCLC clinical samples from ICIs responders and non‐responders showed that high Lgmn expression corresponded to diminished immune responsiveness, whereas low expression was associated with improved immune response to ICIs. (Figure 3J,K), suggesting that Lgmn levels are significantly correlated with immunotherapy prognosis in NSCLC patients. Additionally, we observed that Lgmn expression positively correlated with PD‐1 but negatively correlated with CD8 and GZMB levels (Figure 3L–N), further supporting its potential role in impairing CD8^+^ T cell function and suppressing anti‐tumor immunity in NSCLC. Collectively, these findings suggest that Lgmn^+^ macrophages may contribute significantly to immunotherapy resistance in EGFR‐TKI‐resistant lung cancer.

### 2.4 Macrophage‐Specific Lgmn Deletion Reactivates CD8^+^ T Cell‐Mediated Anti‐Tumor Immunity

**FIGURE 4:**
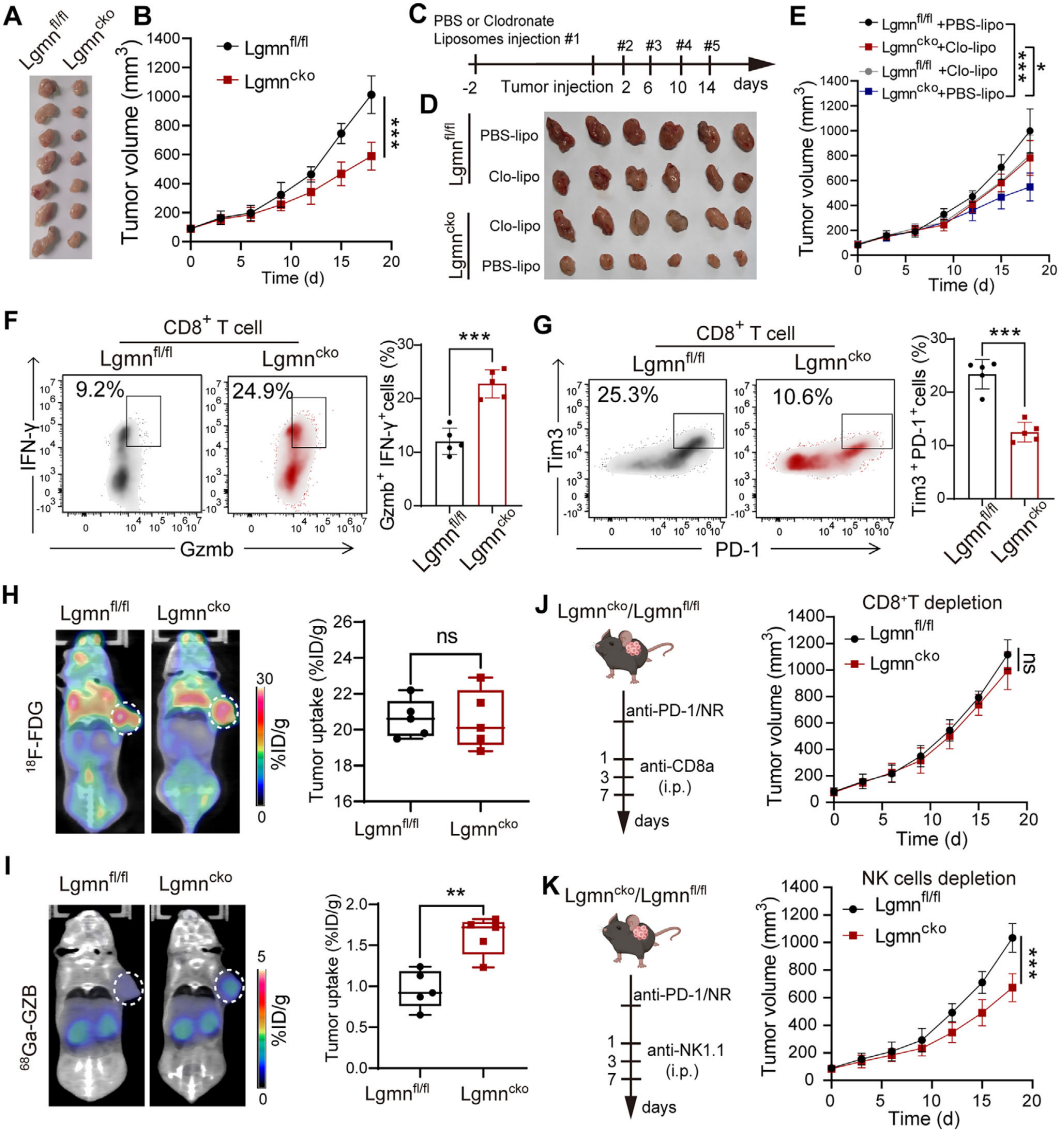
Lgmn deficiency in macrophages enhances CD8^+^ T cell‐mediated antitumor immunity (A, B). Representative photographs (A) and growth curves (B) of tumors from anti‐PD‐1/NR tumor‐bearing Lgmn^fl/fl^ and Lgmn^cko^ mice (n = 7/group). (C) Schematic of Lgmn^fl/fl^ and Lgmn^cko^ mice (n = 6/group) treated with liposomal clodronate. (D‐E) Representative photographs (D) and growth curves (E) of tumors from anti‐PD‐1/NR tumor‐bearing Lgmn^fl/fl^ and Lgmn^cko^ mice treated with liposomal clodronate. (n = 7/group). (F, G) Tumor‐infiltrating Gzmb^+^ IFN‐γ^+^ (F) and TIM‐3^+^ PD‐1^+^ (G) CD8^+^ T cells from anti‐PD‐1/NR tumor‐bearing Lgmn^fl/fl^ and Lgmn^cko^ mice were detected by flow cytometry. (H, I) ^18^F‐FDG (H) and ^68^Ga‐GZB (I) PET imaging of Lgmn^fl/fl^ and Lgmn^cko^ mice (n = 5/group), with representative images on the left and statistical results on the right. (J) Schematic of Lgmn^fl/fl^ and Lgmn^cko^ mice (n = 6/group) treated with anti‐CD8a (left) and the tumor growth of Lgmn^fl/fl^ and Lgmn^cko^ mice (right). (K) Schematic of Lgmn^fl/fl^ and Lgmn^cko^ mice (n = 6/group) treated with anti‐NK1.1 (left) and the tumor growth of Lgmn^fl/fl^ and Lgmn^cko^ mice (right).

To examine the regulatory role of Lgmn^+^ macrophages in anti‐tumor immunity, we crossed Lgmn^fl/fl^ mice with lysozyme 2‐Cre (Lyz2^Cre^) mice to generate Lgmn^fl/fl^ Lyz2^Cre^ mice (hereafter, Lgmn^cko^) (Figure S4A) and transplanted the anti‐PD‐1/NR tumors into syngeneic Lgmn^fl/fl^ and Lgmn^cko^ mice. Notably, Lgmn^cko^ mice exhibited significantly reduced tumor volume and weight compared to Lgmn^fl/fl^ mice (Figure 4A,B), suggesting that loss of Lgmn in tumor‐associated macrophages (TAMs) restrains tumor progression. In addition, systemic macrophage depletion using clodronate liposomes entirely abrogated these protective effects (Figure 4C–E), confirming that tumor suppression was intrinsically dependent on Lgmn deficiency within the TAMs.

We further assessed the role of Lgmn^+^ macrophages in regulating CD8^+^ T cell function. Analysis of tumor‐infiltrating CD8^+^ T cells revealed that Lgmn^cko^ mice had a significantly higher proportion of IFN‐γ^+^ Gzmb^+^ CD8^+^ T cells and a marked reduction in PD‐1^+^ Tim‐3^+^ CD8^+^ T cells compared to Lgmn^fl/fl^ mice (Figure 4F,G). We also observed a substantial increase in the frequency of CD62L^lo^ CD44^hi^ effector memory CD8^+^ T cells within tumors of Lgmn^cko^ mice (Figure S4B). In vivo micro‐PET imaging revealed no difference in ^18^F‐FDG uptake (Figure 4H) but significantly enhanced ^68^Ga‐GZB accumulation in Lgmn^cko^ mice (Figure 4I), providing macroscopic evidence that Lgmn deletion in TAMs reactivates immune responses in EGFR‐TKI‐resistant tumors. To determine whether this effect is primarily mediated by CD8^+^ T cells, we selectively depleted two major immune cell populations involved in antitumor immunity—CD8^+^ T cells (Figure 4J) and NK cells (Figure 4K). CD8^+^ T cell depletion reversed the anti‐tumor effects mediated by Lgmn‐deficient macrophages (Figure 4J), whereas NK cell depletion had no effect on tumor growth inhibition in Lgmn^cko^ mice (Figure 4K). Collectively, these results demonstrate that Lgmn^+^ macrophages suppress CD8^+^ T cell‐mediated anti‐tumor immunity.

### 2.5 Lgmn^+^ Macrophages Regulate CD8^+^ T Cell Dysfunction Through Lgmn‐Containing EVs

**FIGURE 5:**
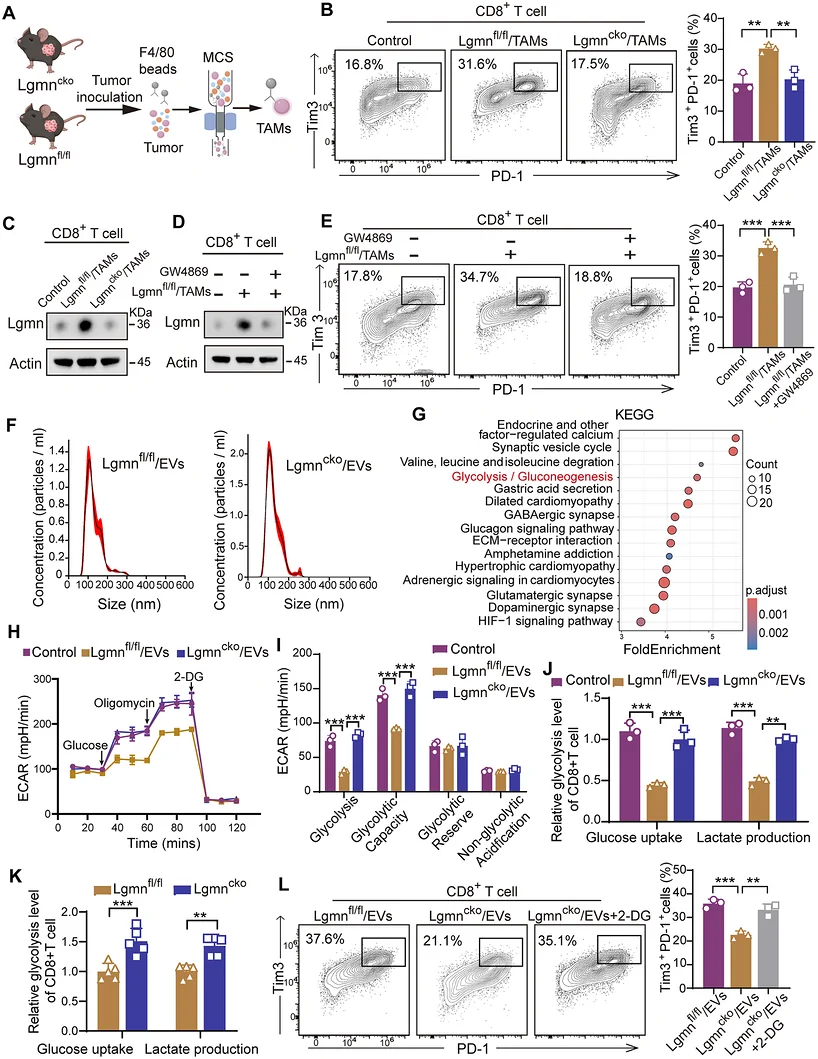
Lgmn^+^:macrophage‐derived EVs induce CD8^+^ T cell exhaustion through glycolytic suppression. (A) Schematic diagram of Lgmn^fl/fl^‐TAMs and Lgmn^cko^‐TAMs extraction. (B) Flow cytometry analysis of TIM‐3^+^ and PD‐1^+^ proportions in CD8^+^ T cells co‐cultured with Lgmn^fl/fl^‐TAMs and Lgmn^cko^‐TAMs at a 1:1 ratio. (C) Western blot analysis of Lgmn expression in CD8^+^ T cells conditioned by Lgmn^fl/fl^‐TAMs and Lgmn^cko^‐TAMs. (D) Western blot analysis of Lgmn expression in CD8^+^ T cells conditioned by Lgmn^fl/fl^‐TAMs and GW4869‐treated Lgmn^fl/fl^‐TAMs. (E) Flow cytometry detection of the TIM‐3^+^ and PD‐1^+^ proportions of CD8^+^ T cells conditioned with Lgmn^fl/fl^‐TAMs and GW4869‐treated Lgmn^fl/fl^‐TAMs. (F) NTA detection of the particle size of Lgmn^fl/fl^/EVs (left) and Lgmn^cko^/EVs (right). (G) KEGG analysis of differentially expressed proteins in CD8^+^ T cells treated with Lgmn^fl/fl^/EVs and Lgmn^cko^/EVs. (H‐I) Seahorse assay of glycolytic activity in CD8^+^ T cells treated with Lgmn^fl/fl^/EVs and Lgmn^cko^/EVs. (J) Detection of glucose uptake and lactate production in CD8^+^ T cells treated with Lgmn^fl/fl^/EVs and Lgmn^cko^/EVs. (K) Detection of glucose uptake and lactate production in tumor‐infiltrating CD8^+^ T cells from Lgmn^fl/fl^ and Lgmn^cko^ mice. (L) Flow cytometry analysis of the TIM‐3^+^ and PD‐1^+^ proportions of CD8^+^ T cells treated with Lgmn^fl/fl^/EVs, Lgmn^cko^/EVs, or 2‐DG.

To elucidate the molecular mechanisms by which Lgmn^+^ macrophages regulate CD8^+^ T cell dysfunction, TAMs were isolated from Lgmn^fl/fl^ and Lgmn^cko^ mice (Figure 5A), and co‐cultured with CD8^+^ T cells. Lgmn^fl/fl^‐TAMs markedly upregulated exhaustion markers PD‐1 and Tim‐3 in CD8^+^ T cells, whereas Lgmn^cko^‐TAMs lost this capacity (Figure 5B). Intriguingly, we detected significant accumulation of Lgmn protein within the CD8^+^ T cells treated with Lgmn^fl/fl^‐TAMs but not Lgmn^cko^‐TAMs (Figure 5C), despite no intrinsic Lgmn transcription (Figure S5A), suggesting that Lgmn^+^ TAMs may transfer Lgmn to modulate CD8^+^ T cell exhaustion. Given that extracellular vesicles (EVs) represent a major vehicle for protein transfer, we blocked EVs secretion using GW4869, which completely prevented both Lgmn transfer (Figure 5D) and the subsequent induction of CD8^+^ T cell exhaustion (Figure 5E), supporting the hypothesis that Lgmn^+^ TAMs promote CD8^+^ T cell exhaustion through EVs‐mediated Lgmn transfer.

We next purified EVs from Lgmn^fl/fl^‐TAMs (Lgmn^fl/fl^/EVs) and Lgmn^cko^‐TAMs (Lgmn^cko^/EVs). Transmission electron microscopy (TEM) revealed spherical particles of approximately 100 nm in diameter with characteristic bilayered membranes (Figure S5B). Nanoparticle tracking analysis (NTA) confirmed their size distribution within the typical range (50–150 nm) (Figure 5F). Lgmn was detected predominantly in the same fraction as known EVs markers CD63 and TSG101 (Figure S5C). Furthermore, we treated cells with Triton X‐100 for permeabilization and Proteinase K (PK) for protein degradation. Lgmn was protected from degradation when only PK was added, indicating that Lgmn is localized inside Lgmn^fl/fl^/EVs as a cargo. Next, we directly treated CD8^+^ T cells with EVs, Lgmn^fl/fl^/EVs, but not Lgmn^cko^/EVs, increased intracellular Lgmn levels (Figure S5D) and exacerbated exhaustion in CD8^+^ T cells (Figure S5E). These results indicate that EVs‐mediated Lgmn transfer is a key mediator of CD8^+^ T cell dysfunction.

### 2.6 Lgmn^+^ Macrophage‐Derived EVs Induce CD8^+^ T Cell Exhaustion Through Glycolytic Suppression

To explore how Lgmn^fl/fl^/EVs mediate CD8^+^ T cell exhaustion, we performed LC‐MS/MS proteomic profiling on CD8^+^ T cells treated with Lgmn^fl/fl^/EVs or Lgmn^cko^/EVs. Using the iBAQ algorithm for absolute protein quantification, 250 proteins were found to be differentially expressed between the two groups (Figure S5F). KEGG pathway analysis revealed significant enrichment of glycolytic pathways in CD8^+^ T cells following Lgmn^fl/fl^/EVs treatment (Figure 5G). Next, we investigated whether Lgmn^fl/fl^/EVs regulate the glycolysis level of CD8^+^ T cells. Extracellular acidification rate (ECAR) measurements demonstrated that Lgmn^fl/fl^/EVs significantly impaired glycolytic capacity in CD8^+^ T cells compared with Lgmn^cko^/EVs (Figure 5H,I). Consistently, Lgmn^fl/fl^/EVs reduced CD8^+^ T cells' glucose uptake and lactate production (Figure 5J). Furthermore, tumor‐infiltrating CD8^+^ T cells from Lgmn^fl/fl^ mice exhibited lower glycolytic activity than those from Lgmn^cko^ mice (Figure 5K). Crucially, glycolytic inhibition with 2‐DG abolished the protective effects of Lgmn^cko^/EVs against T cell exhaustion (Figure 5L). These data demonstrate that Lgmn^fl/fl^/EVs induce CD8^+^ T cell exhaustion through glycolytic suppression.

### 2.7 Lgmn Interacts With and Promotes Eno1 Ubiquitination and Degradation

**FIGURE 6:**
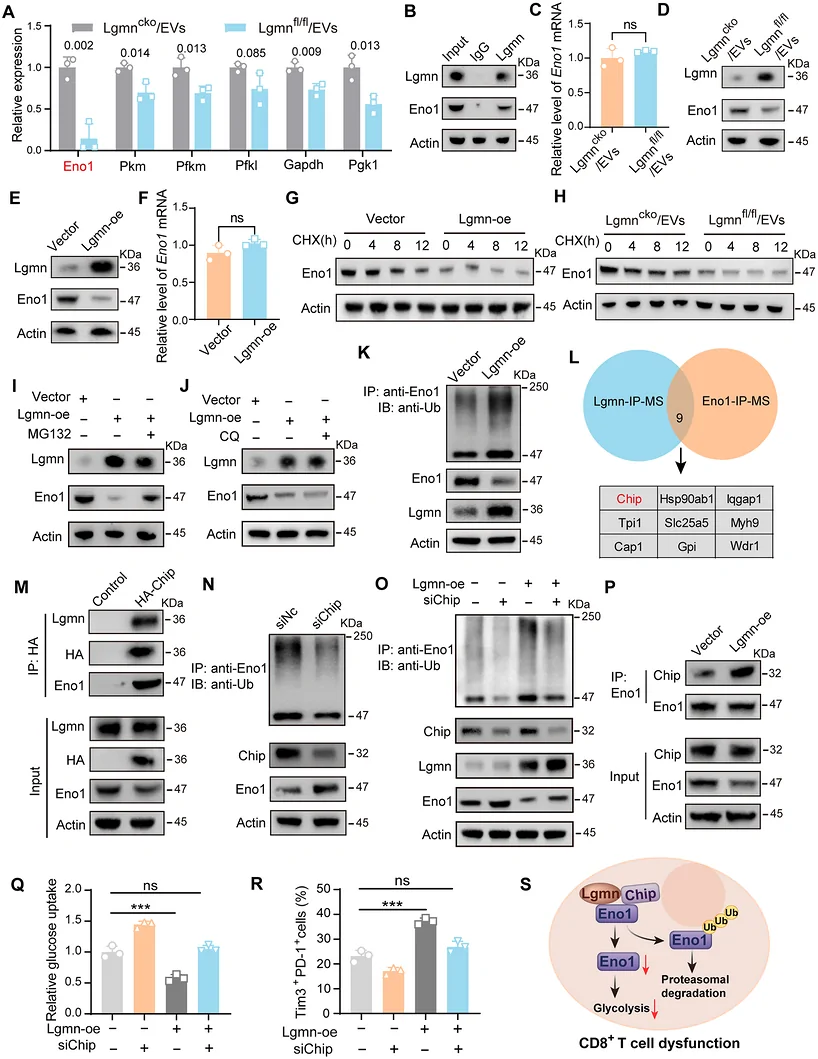
Lgmn recruits Chip to ubiquitinate Eno1 to regulate the dysfunction of CD8^+^ T cells. (A) The expression of glycolysis‐related enzymes in CD8^+^ T cells treated with Lgmn^fl/fl^/EVs and Lgmn^cko^/EVs from proteomics analysis by LC‐MS/MS. (B) Cell lysates were immunoprecipitated using anti‐Lgmn antibodies, and Eno1 expression was detected by Western blotting. (C, D) Eno1 expression in CD8^+^ T cells treated with Lgmn^fl/fl^/EVs and Lgmn^cko^/EVs was detected by qRT‐PCR (C) and Western blotting (D). (E, F) Detection of Eno1 expression in CD8^+^ T cells overexpressing Lgmn by immunoblotting (E) and qRT‐PCR (F). (G, H) Detection of Eno1 protein stability in CD8^+^ T cells overexpressing Lgmn (G) or treated with EVs (H) by immunoblotting. (I) Detect Eno1 expression in CD8^+^ T cells overexpressing Lgmn or treated with MG132 using Western blotting. (J) Detect Eno1 expression in CD8^+^ T cells overexpressing Lgmn or treated with CQ using Western blotting. (K) Detect Eno1 ubiquitination in CD8^+^ T cells overexpressing Lgmn using Western blotting. (L) Cell lysates were immunoprecipitated using anti‐Lgmn or anti‐Eno1 antibodies, and the proteins interacting with Lgmn and Eno1 were detected by LC‐MS. (M) Co‐IP assays were conducted to observe the interaction between Lgmn, Chip, and Eno1. (N) Chip was knocked down, and Eno1 ubiquitination in CD8^+^ T cells was detected by Western blotting. (O) Chip was knocked down, and Lgmn was overexpressed; Eno1 expression and ubiquitination in CD8^+^ T cells were detected by Western blotting. (P) Cell lysates were immunoprecipitated using anti‐Eno1 antibodies, and Chip expression was detected by Western blotting. (Q, R) Chip was knocked down, and Lgmn was overexpressed; glucose uptake in CD8^+^ T cells was detected (Q), and TIM‐3^+^ and PD‐1^+^ CD8^+^ T cells were detected by flow cytometry (R). (S) Illustration of Lgmn enhancing the interaction between Chip and Eno1, and subsequently modulating CD8^+^ T cell exhaustion through metabolic reprogramming.

To elucidate how Lgmn^fl/fl^/EVs inhibit glycolysis in CD8^+^ T cells, we analyzed proteomic data from CD8^+^ T cells treated with Lgmn^fl/fl^/EVs and found differential downregulation of glycolytic enzymes in CD8^+^ T cells, with the most pronounced reduction observed for Eno1 (Figure 6A), suggesting that Lgmn‐containing EVs may modulate glycolysis in CD8^+^ T cells by regulating Eno1 expression. To verify the Lgmn‐Eno1 interaction, we performed immunoprecipitation using an anti‐Lgmn antibody in CD8^+^ T cells treated with Lgmn^fl/fl^/EVs and confirmed the interaction between Lgmn and Eno1 (Figure 6B). In addition, exogenous co‐immunoprecipitation (Co‐IP) assays in HEK‐293T cells co‐transfected with Lgmn and Eno1 further verified this interaction (Figure S6A). Next, we sought to explore the regulatory role of Lgmn on the expression of Eno1. Intriguingly, Lgmn^fl/fl^/EVs had no impact on the *Eno1* mRNA levels (Figure 6C). However, Eno1 protein levels were significantly decreased upon Lgmn^fl/fl^/EVs treatment (Figure 6D). Consistently, Lgmn overexpression suppressed Eno1 protein but not *Eno1* mRNA expression (Figure 6E,F). Next, we treated CD8^+^ T cells with the protein synthesis inhibitor cycloheximide (CHX) for different time periods to examine Eno1 protein stability. Interestingly, Lgmn shortened the half‐life of the Eno1 protein (Figure 6G), corroborated by reduced Eno1 stability in Lgmn^fl/fl^/EVs‐treated cells (Figure 6H). Next, we investigated how Lgmn facilitates Eno1 protein degradation. CD8^+^ T cells overexpressing Lgmn were treated with either the proteasome inhibitor MG132 or the lysosome inhibitor chloroquine (CQ) for 8 h. Notably, MG132 (Figure 6I) but not CQ (Figure 6J) blocked Lgmn‐mediated Eno1 degradation, suggesting that Lgmn regulates Eno1 expression at the post‐translational level in a ubiquitination‐proteasome‐dependent manner.

### 2.8 Lgmn Recruits Chip to Enhance Eno1 Ubiquitination and Promote CD8^+^ T Cell Exhaustion

We next used western blotting to examine ubiquitination levels and found that Lgmn overexpression significantly enhanced Eno1 ubiquitination (Figure 6K). These data demonstrate that Lgmn interacts with Eno1 and promotes its degradation via the ubiquitin‐proteasome pathway. However, as Lgmn does not belong to the family of ubiquitin ligases or deubiquitinating enzymes, we hypothesized that a ubiquitin ligase or deubiquitinating enzyme may mediate this process. To identify potential interactors between Lgmn and Eno1, we performed immunoprecipitation followed by mass spectrometry, which yielded nine candidate proteins. Notably, Chip was the only member of the ubiquitin ligase family among them (Figure 6L). Subsequent co‐IP experiments confirmed that Chip binds to both Lgmn and Eno1 (Figure 6M). We then investigated whether Chip participates in Lgmn‐mediated Eno1 ubiquitination. Notably, knockdown of Chip increased Eno1 protein levels and decreased its ubiquitination (Figure 6N). Furthermore, Chip knockdown significantly reversed the ubiquitination and downregulation of Eno1 caused by Lgmn overexpression in CD8^+^ T cells (Figure 6O). Collectively, these results indicate that Lgmn promotes the degradation of Eno1 by enhancing Chip‐mediated ubiquitination of Eno1.

Next, we asked how Lgmn interacts with Chip to promote Eno1 ubiquitination. Unexpectedly, Lgmn overexpression did not affect Chip expression levels (Figure S6B), suggesting that Lgmn is unlikely to regulate Chip expression. Notably, the Chip‐Eno1 interaction was markedly enhanced upon Lgmn overexpression, indicating that Lgmn facilitates Eno1 ubiquitination by recruiting Chip to bind Eno1 (Figure 6P). Finally, we evaluated the role of Chip inhibition in regulating CD8^+^ T cell function. Chip knockdown significantly reversed Lgmn‐mediated suppression of glycolysis in CD8^+^ T cells (Figure 6Q and Figure S6C) and consequently attenuated CD8^+^ T cell exhaustion (Figure 6R). These data demonstrate that Lgmn recruits Chip to enhance Eno1 ubiquitination, thereby modulating CD8^+^ T cell exhaustion through metabolic reprogramming (Figure 6S).

### 2.9 Targeted Lgmn Treatment Enhances the Efficacy of PD‐1 Blockade

**FIGURE 7:**
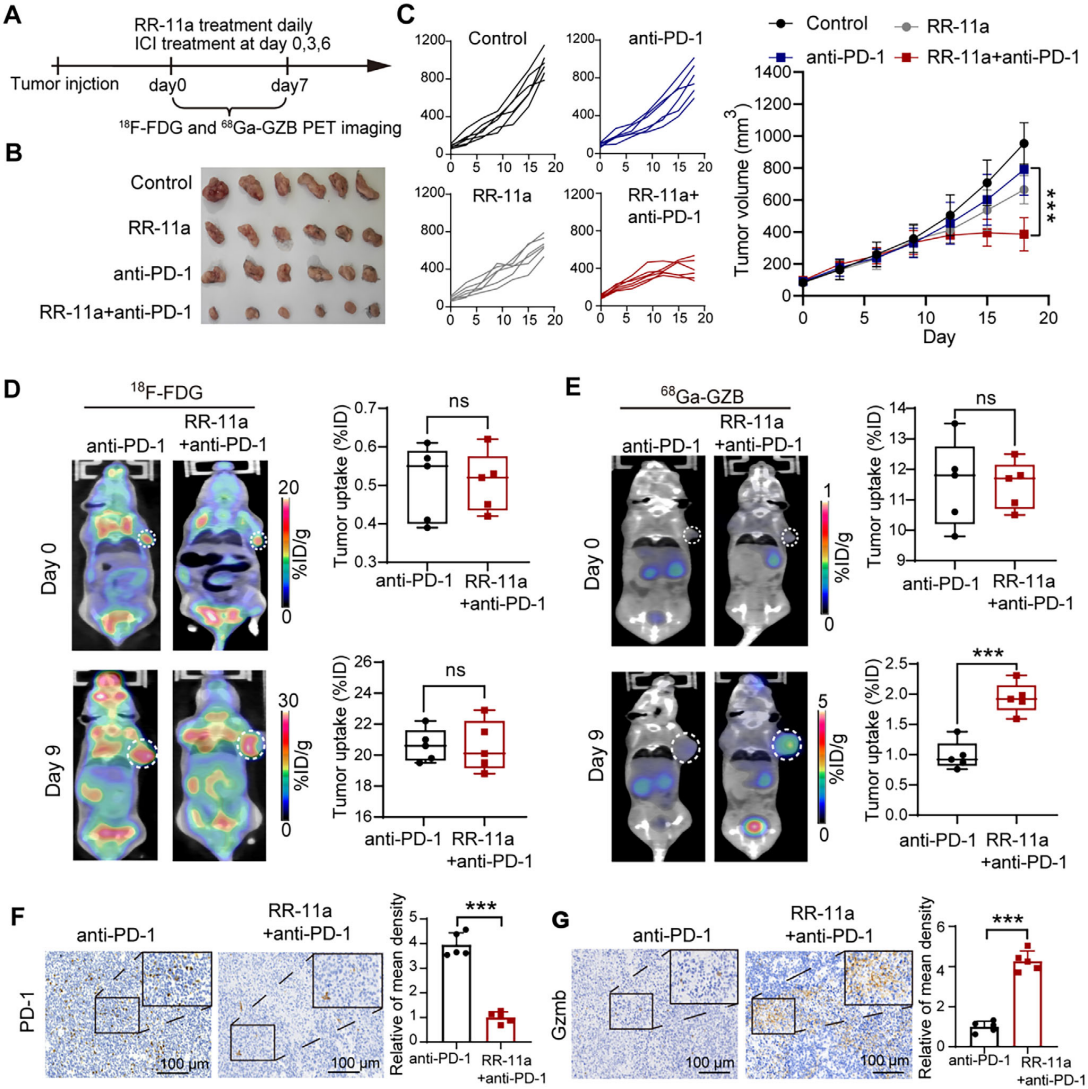
Targeting Lgmn enhances the efficacy of PD‐1 blockade in EGFR‐TKI‐resistant lung cancer. (A) Schematic of the combination of RR‐11a and anti‐PD‐1 therapy for anti‐PD‐1/NR lung cancer with ^18^F‐FDG and ^68^Ga‐GZB PET imaging. (B, C) Tumor growth of anti‐PD‐1/NR tumor‐bearing mice treated with control, anti‐PD‐1 mAb, RR‐11a, or the anti‐PD‐1 mAb combined with RR‐11a (n = 6/group). (D‐E) ^18^F‐FDG (D) and ^68^Ga‐GZB (E) PET imaging of mice treated with anti‐PD‐1 mAb or the anti‐PD‐1 mAb combined with RR‐11a on day 0 (upper) and day 9 (lower) (n = 5/group), with representative images on the left and statistical results on the right. (F, G) The expression of PD‐1 (F) and Gzmb (G) in tumor tissues was detected by immunohistochemistry.

In this study, we found that Lgmn deletion not only inhibited tumor growth but also remodeled the immune landscape of tumors, suggesting that blocking Lgmn may synergize with anti‐PD‐1 treatment. We therefore evaluated the combination of the low‐dose Lgmn inhibitor RR‐11a (20 mg/kg) with anti‐PD‐1 therapy in a preclinical model (Figure 7A). We observed synergistic effects of RR‐11a when combined with anti‐PD‐1 in mice bearing anti‐PD‐1/NR tumors (Figure 7B,C). This combination synergistically enhanced anti‐tumor efficacy in mice bearing EGFR‐TKI‐resistant tumors (Figure 7B,C), outperforming either monotherapy. Moreover, micro‐PET imaging showed higher ^68^Ga‐GZB uptake in the combination group than in anti‐PD‐1 alone (Figure 7D,E). Immunohistochemical analysis revealed a reduction in exhausted CD8^+^ T cells (Figure 7F) and an increase in granzyme B release (Figure 7G) following combination therapy, suggesting that Lgmn targeting contributes to immune reactivation and enhanced antitumor activity. Importantly, this combination therapy maintained a favorable safety profile. Compared with the control group, RR‐11a plus PD‐1 blockade did not induce significant body weight loss in normal mice during the treatment period (Figure S7A). Furthermore, serum biochemical analysis confirmed that key hepatic and renal function markers, as well as cardiac enzymes, remained within normal ranges, with no significant differences observed among the groups (Figure S7B). Histological examination via HE staining of major organs also revealed no noticeable pathological changes, tissue damage, or inflammatory infiltration (Figure S7C). Collectively, these results demonstrate that the combination of RR‐11a and PD‐1 blockade yields potent therapeutic efficacy with no systemic toxicity, supporting its potential as a therapeutic strategy for lung cancer following acquired resistance to EGFR‐TKI.

## 3 Discussion

While immune checkpoint blockade has revolutionized the clinical management of advanced NSCLC, predicting therapeutic response and elucidating the mechanisms of treatment failure remain formidable challenges. This is particularly critical for patients with EGFR‐driven NSCLC, who frequently exhibit primary or acquired resistance to both targeted TKI and subsequent immunotherapy [11, 12]. In this study, we successfully addressed this clinical gap by validating a non‐invasive immunosurveillance imaging platform, ^68^Ga‐GZB PET/CT, in a prospective cohort of NSCLC patients. Furthermore, by applying this clinically validated tool to preclinical models of EGFR‐TKI resistance, we found that immune reactivity can be dynamically altered following TKI resistance in a subset of lung cancers. Moreover, we uncovered a novel, targetable immune evasion axis: Lgmn^+^ macrophage‐derived extracellular vesicles deliver Lgmn to CD8^+^ T cells, where it recruits the E3 ubiquitin ligase Chip to mediate ubiquitination and proteasomal degradation of the glycolytic enzyme Eno1, thereby inhibiting glycolysis in CD8^+^ T cells and driving their exhaustion. This work establishes ^68^Ga‐GZB PET/CT as a promising tool for assessing immune reactivity in NSCLC and for stratifying post‐TKI immunotherapy responses. We also delineate the role of Lgmn^+^ macrophages in driving immunotherapy resistance and propose that combining Lgmn‐targeted therapy with immunotherapy represents an innovative treatment strategy for lung cancer patients following EGFR‐TKI treatment failure.

Immune checkpoint inhibitors targeting the PD‐L1/PD‐1 axis can activate the antitumor activity of cytotoxic T cells and improve clinical outcomes in patients with locally advanced or metastatic NSCLC without EGFR or ALK driver mutations [13, 14]. However, patients with EGFR‐mutant NSCLC derive limited benefit from such treatments [14]. Therefore, selecting appropriate treatment strategies for EGFR‐mutant NSCLC patients following resistance to first‐line EGFR‐TKI therapy remains a significant clinical challenge. Previous clinical studies have demonstrated that a subset of patients developing resistance to EGFR‐TKIs through non‐T790M mutation mechanisms may derive clinical benefit from ICI therapy [2]. Furthermore, Isomoto K et al. reported that EGFR‐TKI treatment can remodel the tumor microenvironment of EGFR‐mutant NSCLC and potentially enhance immunogenicity, for example, by upregulating PD‐L1 expression and tumor mutational burden, thereby improving ICI response rates in a subset of patients [3]. These TKI‐induced dynamic alterations render specific NSCLC subpopulations highly sensitive to ICI. However, precisely identifying these potential responders remains a major challenge, and the underlying molecular mechanisms driving these dynamic changes remain elusive.

To bridge this clinical gap, our study highlights the predictive potential of ^6^ ^8^Ga‐GZB PET/CT. Conventional PET tracer ^1^ ^8^F‐FDG reflects tumor glucose metabolism but cannot differentiate tumor cells from immune cells [4]. Because immunotherapy frequently induces peritumoral inflammation and necrosis, ^1^ ^8^F‐FDG PET is prone to false positives and struggles to distinguish pseudoprogression or hyperprogression [15]. Although novel tracers targeting immune markers such as CD4 and CD8 have emerged [16, 17], their predictive value remains limited. Such imaging merely quantifies the abundance of infiltrating lymphocytes without distinguishing cytotoxic effector T cells, immunosuppressive regulatory T cells (Tregs), or exhausted T cells. In contrast, cytotoxic T lymphocytes (CTLs) and natural killer (NK) cells exert anti‐tumor effects primarily by releasing granzyme B, which initiates an enzymatic cascade that induces target cell apoptosis [18]. By directly quantifying granzyme B secretion, ^6^ ^8^Ga‐GZB captures the terminal, functional execution of anti‐tumor immunity. Therefore, ^6^ ^8^Ga‐GZB PET can serve as an imaging platform across various immunotherapy scenarios, such as PD‐1/PD‐L1 and CTLA‐4 inhibitors. In our NSCLC cohort, the correlation between ^6^ ^8^Ga‐GZB uptake and immunotherapy response strongly supports its broad potential as a predictive biomarker. With this imaging tool, we have a unique advantage in noninvasively and dynamically tracking the immune remodeling that occurs during the evolution of EGFR‐TKI resistance, a phenomenon that has previously been difficult to monitor non‐invasively.

The complexity of the immune microenvironment significantly contributes to immunotherapy tolerance, wherein immunosuppressive macrophages function as master regulators by engaging in multifaceted crosstalk with various immune and stromal cells [19, 20]. For instance, macrophages secrete various cytokines and extracellular vesicles to actively modulate the function of other immune cells, including inhibiting T cell activation and cytotoxicity, facilitating Treg differentiation, and impairing dendritic cell antigen presentation [20]. Such multifaceted intercellular communication substantially undermines effective immune surveillance. Our single‐cell and functional analyses of the TKI‐resistant tumor immune microenvironment revealed a highly immunosuppressive niche orchestrated by Lgmn^+^ macrophages. We demonstrate that Lgmn^+^ macrophages package and deliver Lgmn protein directly into CD8^+^ T cells via extracellular vesicles. This EV‐mediated intercellular protein transfer highlights a critical yet underexplored dimension of TIME crosstalk, extending beyond canonical receptor‐ligand interactions to enable direct intracellular reprogramming.

Extracellular vesicles have emerged as essential mediators of intercellular communication, with increasing attention focused on the functional roles of immune cell‐derived extracellular vesicles [21, 22]. Notably, extracellular vesicles carrying immune checkpoint molecules such as PD‐L1 and CTLA‐4 facilitate tumor immune escape [23], while macrophage‐derived extracellular vesicles can deliver NAMPT to modulate nicotinamide (NAM) metabolism in CAFs, thereby sustaining an immunosuppressive tumor microenvironment [24]. In this study, we demonstrate that upon internalization by CD8^+^ T cells, Lgmn does not function as a conventional protease but rather serves as a critical scaffolding protein that recruits the E3 ubiquitin ligase Chip. This pathological recruitment selectively accelerates the ubiquitination and subsequent proteasomal degradation of Eno1, a key enzyme within the glycolytic pathway. The resulting metabolic disruption deprives CD8^+^ T cells of the rapid glycolytic flux essential for effector function, thereby driving them into exhaustion. This process fosters an immunosuppressive TIME conducive to tumor progression. Collectively, our findings uncover a novel mechanism underlying immune escape in EGFR‐TKI‐resistant lung cancer.

Despite these encouraging findings, our study has several limitations. First, the clinical predictive efficacy of ^6^ ^8^Ga‐GZB was evaluated in a single‐center prospective cohort with a limited sample size, and thus its predictive value requires further validation in larger, multicenter studies. Moreover, the limited number of EGFR‐TKI‐resistant patients in our cohort precluded a reliable statistical subgroup analysis at the clinical level to assess how TKI resistance affects ^6^ ^8^Ga‐GZB uptake. Although we partially offset this limitation by establishing preclinical models to explore the underlying mechanisms, future large‐scale clinical cohort studies specifically designed for the EGFR‐TKI‐resistant population remain necessary to definitively validate these findings in patients. Second, granzyme B is also involved in the pathogenesis of various inflammatory diseases, potentially leading to false‐positive uptake signals in non‐tumor tissues. Therefore, future studies should focus on optimizing imaging protocols and interpretation strategies to more accurately differentiate tumor‐specific immune responses from systemic non‐specific inflammation.

In this study, we establish a non‐invasive and precise molecular imaging approach for monitoring early immune responses in NSCLC, clearly revealing the dynamic immune changes following EGFR‐TKI resistance. From the perspective of Lgmn^+^ macrophage‐CD8^+^ T cell crosstalk, we elucidate the mechanism underlying sustained immunotherapy tolerance in NSCLC after EGFR‐TKI resistance. Overall, this study provides clinical guidance for subsequent immunotherapy in EGFR‐TKI‐resistant patients, and innovatively identifies Lgmn‐targeted combination immunotherapy as a novel therapeutic strategy for NSCLC patients following EGFR‐TKI resistance.

## 4 Methods

### 4.1 Clinical Trial Design

The prospective study was approved by the Ethics Committee of Fudan University Shanghai Cancer Center (number 2201249‐28) and registered online at the Chinese Clinical Trial Registry (ChiCTR2200062089). In total, 45 patients with NSCLC who underwent ^68^Ga‐GZB PET/CT imaging at FUSCC between October 2022 and May 2025 were included in this study. The inclusion criteria were as follows: patients diagnosed with NSCLC according to the guidelines of the Chinese Society of Clinical Oncology; patients who had received a complete course of immunotherapy; patients who had received ^68^Ga‐GZB PET/CT in the Department of Nuclear Medicine of our hospital at the mid‐course (2−3 cycles) of the immunotherapy and the patients continued to undergo immunotherapy after imaging, age of at least 18 y. The exclusion criteria for the final analysis were as follows: patients who could not complete full‐course immunotherapy, patients with no measurable target lesions, patients lost to follow‐up, and patients who had other malignant tumors. Patients who met the selection criteria and remained after the exclusion process were included in the final analysis. Patient follow‐up was done by FUSCC physicians; tumor response was assessed by the attending physicians and imaging physicians according to Response Evaluation Criteria in Solid Tumors (RECIST) version 1.1.

### 4.2 PET/CT Imaging

For ^68^Ga‐GZB PET/CT, the injected activity was 148–185 MBq. Scanning was performed 30 min after injection with the PET/CT scanner (Biograph 16 HR; Siemens Medical Systems). The ^68^Ga‐GZB PET/CT images were independently analysed and interpreted by three experienced nuclear medicine physicians, who reached a consensus on inconsistencies. Lesions with increased radioactivity compared to surrounding normal tissue and not accompanied by physiological uptake were considered to be suspected malignant lesions. SUVmax and SUVmean were calculated manually by plotting the three‐dimensional volume of interest of the tumor lesion and normalised to body weight.

### 4.3 Cell Culture

Human embryonic kidney HEK293T cell lines were purchased from the American Type Culture Collection, USA. Mouse lung cancer LLC cell lines were purchased from the Cell Center of the Chinese Academy of Sciences in Shanghai, China. Cell lines were cultured in RPMI‐1640 or DMEM medium (Hyclone, USA) supplemented with 10% FBS and antibiotics, at 37°C.

The primary mouse TAMs were isolated as follows. The tumors were mechanically dissociated and subjected to digestion with collagenase type IV (1 mg/mL) (MilliporeSigma) and DNase I (100 µg/mL) (MilliporeSigma) at 37°C for 60 min. The resulting cell suspension was then filtered using a 70 µm cell strainer. Resuspend the red blood cells with 2 mL of red blood cell lysis buffer and incubate for 2 min at room temperature. According to the manufacturer's instructions, TAMs were sorted by F4/80 positive magnetic sorting (PE anti‐mouse F4/80 Recombinant Antibody,157304, Biolegend). The isolated TAMs were cultured in RPMI‐1640 medium supplemented with 10% FBS.

Peripheral blood mononuclear cells (PBMCs) were first isolated by density gradient centrifugation using lymphocyte separation medium. CD8^+^ T cells were then specifically purified using the Mouse CD8^+^ T Cell Isolation Kit (STEMCELL) according to the manufacturer's protocol. The purified CD8^+^ T cells were co‐cultured with Mouse T‐Activator CD3/CD28 Dynabeads (ThermoFisher) at a 1:1 bead‐to‐cell ratio for 48 h to achieve T cell activation. The resulting activated CD8^+^ T cells were subsequently used for further experimental studies.

### 4.4 Animals

Six‐week‐old wild‐type C57BL/6 mice were purchased from GemPharmatech (Nanjing, China). Lgmn‐flox mice (Strain No. T009982) and Lyz2‐cre mice (Strain NO. T003822) were purchased from GemPharmatech (Nanjing, China). Lgmn^fl/fl^ were crossed with Lyz2‐cre mice to generate Lgmn^cko^ mice. All experimental mice were bred and maintained under specific pathogen‐free conditions. Six to nine‐week‐old mice of both sexes were used for the experiments. To construct a mouse model of lung cancer, 500,000 cells/100 µl of lung cancer cells were injected subcutaneously into the right axilla of mice, and tumor size was measured every three days. The volume was calculated using the formula V = (width by width by length)/2.

For cancer immunotherapy, each mouse was injected intraperitoneally with anti‐PD‐1 (200 µg per mouse; BioXcell, BE0146) every three days. For cancer EGFR‐TKI treatment, AZD9291 (5 mg/kg) was administered orally daily to each mouse. For cancer Lgmn inhibitor treatment, RR‐11a (20 mg/kg) was injected intraperitoneally daily to each mouse. All in vivo experiments were carried out in accordance with the requirements of the Animal Research Committee of Fudan University on the care and use of experimental animals in research (FUSCC‐IACUC‐S2022‐0565). Our study examined male and female animals, and similar findings are reported for both sexes.

### 4.5 Clinical Samples

Specimens were obtained from 13 patients who underwent surgery (with subsequent immunotherapy) from the surgical specimen archive of the Shanghai Cancer Prevention and Control Centre of Fudan University. Ethical approval was granted by the ethics committee of Fudan University Shanghai Cancer Center.

### 4.6 ^68^Ga‐GZB and ^18^F‐FDG Micro‐PET/CT

Mice were subjected to a 6‐h fasting period prior to the administration of ^18^F‐FDG via the tail vein, whereas no fasting regimen was implemented before the injection of the ^68^Ga‐GZB tracer. Anesthesia for mice harboring lung cancer tumors was induced using a blend of oxygen and isoflurane gas (1%‐2.5% isoflurane). Subsequent FDG or GZB micro‐PET/CT scans were conducted 60 min post‐administration of the respective tracers at doses ranging from 150–200 µCi. Following the CT scans, a 10‐min PET acquisition was promptly carried out utilizing the Siemens Inveon PET/CT system. Image analysis was performed utilizing the Inveon Research Workplace 4.2 software, with regions of interest delineated to quantify tumor uptake.

### 4.7 Flow Cytometry

To quantify tumor‐infiltrating lymphocytes and the expression of effector molecules in these cells, the tumors were mechanically dissociated and subjected to digestion with collagenase type IV (1 mg/mL) (MilliporeSigma) and DNase I (100 µg/mL) (MilliporeSigma) at 37°C for 60 min. The resulting cell suspension was then filtered using a 70 µm cell strainer. Block filtered cells with anti‐CD16/32 antibody (101320, BioLegend, 1:50), and dead cells were labeled using live/dead dye (423102, BioLegend). To quantify the infiltration of cytotoxic T lymphocyte, anti‐CD45 (103134, biolegend, 1:100), anti‐CD3 (100306, biolegend, 1:100), anti‐CD8a (100712, biolegend, 1:100), anti‐IFN‐γ (505826, biolegend, 1:50), anti‐Granzyme B (372208, biolegend, 1:50), anti‐PD‐1(135205, biolegend, 1:100) and anti‐Tim‐3 (134009, biolegend, 1:100) antibodies were added into the cell suspension. All samples were run on a Beckman Coulter CytoFLEX and analyzed by FlowJo (version 10.8.1, Tree Star).

### 4.8 RNA Extraction and Quantitative Real‐Time PCR (qRT‐PCR) Analysis

Total RNA was extracted from cells or tissues using TRIzol reagent (Thermo Fisher Scientific, USA). According to the manufacturer's instructions, the quality and quantity of extracted RNA were evaluated by a NanoDrop 2000 spectrophotometer (Thermo Fisher Scientific, USA). The cDNA was synthesized from mRNA by HiScript II Q RT SuperMix (Vazyme, Nanjing, China) under the recommended conditions. Real‐time PCR was performed using an SYBR Green RT‐PCR kit (Vazyme, Nanjing, China) on QuantStudioTM 7 Flex (Thermo Fisher Scientific). Gene expression was quantified using the 2‐∆Ct method. Primer sequences were as follows: Cyclophilin B forward, 5′‐AGATGTAGGCCGGGTGATCT‐3′ and reverse, 5′‐ CCGCCCTGGATCATGAAGTC ‐3′. Eno1 forward, 5′‐TGCGTCCACTGGCATCTAC‐3′ and reverse, 5′‐CAGAGCAGGCGCAATAGTTTTA‐3′.

### 4.9 Histological Analyses

Tumor tissues were fixed with 4% paraformaldehyde and embedded in paraffin. Multicolour immunofluorescence was performed using the Opal 4‐colour manual IHC assay kit (abs50012, Absin) according to the manufacturer's protocol. Sections were antigenically repaired, closed, and incubated with primary antibody for 1 h. A peroxidase‐labelled goat anti‐rabbit/mouse secondary antibody was used and stained with fluorescent dye. For multiple fluorescent staining, sections were subjected to an antigen repair step to remove bound antibodies and then incubated with another primary antibody. This was repeated until all antigens were stained. The following antibodies were used: anti‐Lgmn (A23776, ABclonal), anti‐CD8a (#D8A8Y, Cell Signaling Technology), anti‐PD‐1 (#84651, Cell Signaling Technology), and anti‐Granzyme B (#46890, Cell Signaling Technology). Finally, sections were counterstained with DAPI and mounted with glycerol and gelatin mounting medium. Tissue sections were imaged using an A1 scanning confocal microscope (Nikon), and image data were collected using NIS Elements (v4.50.00, Nikon).

### 4.10 Single‐Cell Sequencing

The mouse seed tumor tissue was cut into 0.5 g pieces, and the enzyme mixture (4.7 mL RPMI 1640, 200 µL Enzyme H, 100 µL Enzyme R, and 25 µL Enzyme) was configured. The tumor pieces were placed in the enzyme mixture and incubated at 37°C in a constant‐temperature shaker for 40 min. After 80‐mesh cell sieve filtration, erythrocyte lysate and dead cell removal reagent were added, and the cells were extracted for reverse transcription; single‐cell libraries were prepared according to standard protocols, and paired‐end sequencing was performed using the Illumina NovaSeq 6000 system.

### 4.11 Reduction of Dimensions, Clustering, and Visualization Techniques

In each sample dataset, cell subsets were identified from a filtered expression matrix. Clustering was executed with Seurat. To normalize the filtered gene expression matrix, Seurat's NormalizeData function was used: each gene's UMI count was divided by the total UMIs per cell, scaled by 10,000, and transformed into a logarithmic scale (ln(UMI‐per‐10000+1)). Highly variable genes were then pinpointed and employed in Principal Component Analysis (PCA). Following this, clustering utilized graph‐based methods, while visualization was achieved through t‐Distributed Stochastic Neighbor Embedding (t‐SNE) or Uniform Manifold Approximation and Projection (UMAP) using Seurat's RunTSNE and RunUMAP functions. To combine multiple samples, we used Harmony integration, a method that minimizes technical batch effects while retaining biological variability during multi‐batch integration. RunHarmony outputs a Seurat object with adjusted Harmony coordinates. Re‐clustering was performed on this modified manifold, using Harmony embeddings instead of Principal Components (PCs), by setting the ‘reduction’ parameter to ‘harmony’ along with other Seurat analysis settings.

### 4.12 Development Trajectory and Pseudotime Analysis

Pseudotime analysis and visualization were conducted using dyno 0.1.2. Initially, the integrated expression data for myeloid cells from the Seurat object was extracted with the “wrap_expression” function. Following this, the developmental trajectory was deduced using the “infer_trajectory” function, employing the “slingshot” method.

### 4.13 Preparation of Bulk RNA Public Datasets

Bulk RNA‐sequencing data and corresponding clinical annotations for NSCLC were obtained from publicly available databases. Among them, the Cancer Genome Atlas (TCGA) cohort used in this study is the lung adenocarcinoma (TCGA‐LUAD) dataset, which exclusively comprises LUAD patients, while the GSE161537 cohort includes LUAD patients who received ICI therapy.

### 4.14 Cellular Communications

Intercellular signaling among immune cell subsets was also inferred using the CellChat (v1.1.3) framework, which evaluates ligand‐receptor interactions. Normalized Seurat data were converted into a CellChat object, and the CellChatDB.mouse database was applied as the reference for ligand‐receptor pairs. Communication probabilities were then estimated with the computeCommunProb function, allowing visualization of cellular crosstalk in terms of both interaction frequency and interaction strength.

### 4.15 TAMs, NK or CD8^+^ T Cells Depletion Assay

CD8^+^ T cells and NK cells were depleted by intraperitoneally (i.p.) administering anti‐CD8β (100 µg/mouse) (#BE0061, BioXcell) and anti‐NK1.1 (100 µg/mouse) (#BE0036, BioXcell) on days −1, 3, and 7, respectively. Liposome clodronate (1 mg/mouse on days −1, 5, and 10) (#40337ES, Yeasen) was used to remove phagocytic cells in mice, and plain liposomes were used as a control.

### 4.16 Cell Transfection

Specific siRNAs targeting Chip and corresponding negative control siRNAs (siNC) were purchased from Tsingke (Beijing, China). The siRNAs were transfected into cells using Lipofectamine 8000 transfection reagents (Beyotime) following the manufacturer's instructions. Knockdown efficiency was determined by immunoblotting analysis after 48 h of transfection. The Chip siRNA target sequence: CCCTTCGCATTGCTAAGAAGA.

### 4.17 Western Blotting

Cells were lysed using radioimmunoprecipitation assay (RIPA) buffer (KGP704, Keygenbio) containing protease inhibitor cocktail (P8340, Sigma–Aldrich), and total protein concentration was quantified using a BCA assay kit (Thermo Fisher Scientific). Experiments followed the published protocol from Abcam. Primary antibodies used are as follows: anti‐β‐actin (#4970, Cell Signaling Technology, 1:2000), anti‐Lgmn (A23776, ABclonal, 1:1000), anti‐Eno1 (A11448, ABclonal, 1:1000), anti‐Chip (#2080, Cell Signaling Technology, 1:1000), anti‐HA (AE105, ABclonal, 1:20000), anti‐FLAG (AE005, ABclonal, 1:10000), anti‐Ubiquitin (A19686, ABclonal, 1:1000). Images were detected by the bioanalytical imaging system.

### 4.18 Immunoprecipitation (IP) and Co‐Immunoprecipitation (co‐IP)

Cells were lysed using RIPA lysate (Thermo Fisher) containing protease and phosphatase inhibitors. After incubation with the indicated antibodies for 24 h, and then incubated with protein A/G (Thermo Fisher Scientific) for 3 h. For tagged proteins, we used immunomagnetic beads conjugated to anti‐Flag/anti‐HA antibodies. After IP, protein A/G agarose or magnetic beads were washed three times with TBST and then eluted with SDS lysis buffer, followed by Western blotting.

### 4.19 Protein Stability Assay

To detect protein stability, Cells were treated with 150 µm cycloheximide (CHX, MCE) to block protein synthesis. Cells were harvested after CHX treatment for different times. Western blot analysis was performed as described previously to observe and quantify protein levels.

### 4.20 Glucose Consumption and Lactate Production

CD8^+^ T cells were cultured and treated under the indicated conditions. At the end of the experiment, the culture medium was collected to assess glucose and lactate levels. Glucose concentration was measured using the Glucose Quantitation Assay Kit  (Sigma–Aldrich, USA), while lactate levels were determined with the Lactate Assay Kit (Jiancheng Bioengineering Institute, China), following the manufacturers' protocols. Glucose consumption was calculated as the difference in glucose concentration between fresh medium and cell supernatant, whereas lactate production was determined as the difference in lactate concentration between the cell supernatant and fresh medium. Both values were normalized to cell number for comparative analysis.

### 4.21 Seahorse Assay

Extracellular acidification rate (ECAR) was measured using the XF96 Extracellular Flux Analyzer (Seahorse Bioscience). Prior to the assay, 20,000 extracellular vesicles ‐pretreated CD8^+^ T cells were seeded per well. Cells were maintained at 37°C in a non‐CO_2_ incubator, and 1 h before measurement, the medium was replaced with XF basal medium supplemented with 1 mmol/L pyruvate, 2 mmol/L glutamine, and 10 mmol/L glucose. During the assay, cells were sequentially treated with 2 µmol/L oligomycin (ATP synthase inhibitor) and 150 mmol/L 2‐deoxyglucose (2‐DG) (glycolysis inhibitor). Following ECAR measurement, cells were washed with PBS, fixed in 3% paraformaldehyde, permeabilized with 0.2% Tween‐20, and counterstained with DAPI (1:500) for cell number quantification. Data were analyzed using Seahorse XF Wave Software (v2.6.0).

### 4.22 Ubiquitination Assay

To assess Eno1 ubiquitination catalyzed by Chip, CD8^+^ T cells were treated with 10 µg/ml MG132 for 8 h prior to harvest for denaturing IP‐WB analysis. Cells were washed once with PBS and lysed directly in denaturing lysis buffer (150 mm NaCl, 0.1% NP‐40/Triton X‐100, 25 mm Tris‐HCl [pH 8.0], 5 mm EDTA, 10% glycerol, and 1% SDS). Lysates were heat‐denatured at 100°C for 20 min to inactivate deubiquitinating enzymes. Processed lysates were then subjected to WB or IP‐WB analysis.

### 4.23 Liquid Chromatography‐Tandem Mass Spectrometry (LC‐MS/MS)

Proteomic Analysis: Samples were cryogenically pulverized in liquid nitrogen to obtain dry powder, followed by protein precipitation with propanol at 4°C for 2 h. The precipitated proteins were subjected to tryptic digestion (37°C, overnight), after which a 100 µg aliquot of the digest was acidified with 0.1% formic acid (FA). The acidified samples were fractionated through Strata‐X C18 columns (Phenomenex, Torrance, CA, USA) using a three‐step loading procedure, with column washing performed using 0.1% FA containing 5% acetonitrile. Peptides were subsequently eluted with 1 mL of 80% acetonitrile/0.1% FA solution. The purified peptides were then analyzed by nanoflow liquid chromatography‐tandem mass spectrometry (nanoLC‐MS/MS) on an AB SCIEX TripleTOF 5600+ system, and the resulting raw MS data were processed using MaxQuant software for comprehensive proteomic analysis.

### 4.24 Extracellular Vesicles Isolation

Extracellular vesicles were purified from cell‐conditioned media through differential ultracentrifugation. Briefly, cell culture supernatants were sequentially filtered through 0.22 µm sterile filters (Millipore) to eliminate cellular debris, then subjected to primary ultracentrifugation at 120 000×g for 90 min at 4°C using an Optima XPN‐100 ultracentrifuge (Beckman Coulter, USA). The resulting extracellular vesicle pellets were washed with ice‐cold PBS and centrifuged again under identical conditions to ensure purity. The final extracellular vesicle preparations were resuspended in sterile PBS, aliquoted, and cryopreserved at ‐80°C for subsequent experiments. Quantitative analysis was performed using the Pierce BCA Protein Assay Kit (Thermo Fisher Scientific, USA) following the manufacturer's instructions to determine protein concentration.

### 4.25 Nanoparticle Tracking Analysis

Extracellular vesicle size distribution and concentration were quantitatively assessed using nanoparticle tracking analysis (NTA) on the ZetaView PMX 110 system (Particle Metrix, Meerbusch, Germany). Prior to analysis, extracellular vesicles were diluted in PBS to achieve optimal particle concentrations (10^7–10^9 particles/mL) for accurate measurement. The instrument's proprietary ZetaView software (version 8.04.02) was employed for data acquisition and analysis.

### 4.26 Transmission Electron Microscopy (TEM)

Extracellular vesicles were fixed with an equal volume of 4% paraformaldehyde and deposited onto formvar‐carbon‐coated copper grids for 20 min at room temperature. Following adsorption, the samples were further fixed with 1% glutaraldehyde in PBS for 5 min, then thoroughly rinsed with deionized water (three washes, 2 min each). For negative staining, the grids were treated with 2% uranyl acetate (w/v) for 10 min at room temperature. After carefully blotting excess stain with filter paper, the samples were air‐dried and subsequently examined using transmission electron microscopy.

### 4.27 Statistical Methods

Expressed as mean ± standard deviation (SD) using GraphPad Prism 8.0.4 software (GraphPad Inc.). Statistical analyses for comparison of the two groups were performed using the two‐tailed Student's t‐test or the nonparametric Mann‐Whitney test. One‐way ANOVA was used to compare data from more than two groups under a single treatment, whereas two‐way ANOVA was used to compare data from more than two groups under multiple treatments. Correlation analysis was performed using Pearson's correlation coefficient. p‐values less than 0.05 were considered statistically significant, denoted as ^*^ *p* < 0.05, ^**^ *p* < 0.01, ^***^ *p* < 0.001.

## Author Contributions

S. S. and D.W. designed and led the project. D.W., W.L., Q.L., J.C., W.T., B.Z. and Q.Z. performed experiments. D.W., W.L., W.T., B.Z., Q.Z., X.X. and S.T. analyzed data. B.Z. and Q.L. provided clinical information. S.S., X.X., and S.T. guided data analysis. D.W., Q.L. and W.T. wrote the manuscript. All authors contributed to data interpretation and discussion of results in the manuscript.

## Conflicts of Interest

The authors declare no conflicts of interest.

## Supporting information




**Supporting File**: advs77837‐sup‐0001‐SuppMat.docx.

## Data Availability

The data that support the findings of this study are available from the corresponding author upon reasonable request.
