# Supplementary material for: Granzyme B PET Imaging Reveals Lgmn+ Macrophage‐Mediated Immune Evasion and Guides Immunotherapy in EGFR‐TKI‐Resistant NSCLC
Source: Adv Sci (Weinh). 2026 Sep 16:e77837. Online ahead of print. doi: 10.1002/advs.77837 (PMC13583114; doi:10.1002/advs.77837)
Supplement: Supplementary file 1 [file ADVS-9999-e77837-s001.docx]

**Supplementary Figures**

**Figure S1. ^68^Ga-GZB PET/CT effectively assesses early immunotherapy response in NSCLC patients.**

(**A**) The synthesis of ^68^Ga-GZB(^68^Ga-NOTA-GGGG-IEPD-CHO). (**B**) Receiver operating characteristic (ROC) curves of ^68^Ga-GZB SUVmax.

**Figure S2. ^68^Ga-GZB PET/CT effectively differentiates different immune responses in EGFR-TKIs-resistant lung cancer models.**

(**A**) Immunohistochemical staining for granzyme B in tumor tissue of EGFR mutant lung cancer mice before and after anti-PD-1 treatment. (**B**) Violin plot showing the 5 basic cell types defined according to the marker gene. (**C**) Cell density plots of these 5 cell types in two groups of tumors (density plots). (**D**) Cell percentage of these 5 cell types in two groups of tumors. (**E**) Cell density plots of T cells and NK cells in two groups of tumors classifying cells according to marker genes. (**F**) UMAP plot demonstrating the classification of T cell subpopulations.

**Figure S3. Immunosuppressive Lgmn^+^ macrophage populations are major constituents of the immune-tolerant EGFR-TKIs-resistant lung cancer microenvironment.**

(**A**) Dot plot demonstrating specific marker expression for myeloid cell populations. (**B**) Lineage tracing analysis of Lgmn^+^ macrophages. (**C**) Immunotherapy response proportions in lung cancer patients according to macrophage Lgmn expression (high/low) in GSE161537.

**Figure S4. Lgmn deficiency in macrophages enhances CD8^+^ T cell-mediated antitumor immunity.**

(A) Based on the structure of the Lgmn gene, exon 3 of the Lgmn-201 (ENSMUST00000021607.8) transcript was used as the knockout region, knockout of which would result in disruption of protein function, and Lgmn-flox mice were mated and bred with Lyz2-cre mice to obtain Lgmn^cko^ mice. (**B**) Flow cytometry analysis of CD62L^+^ and CD44^+^ proportions in tumor-infiltrating CD8^+^ T cells from Lgmn^fl/fl^ and Lgmn^cko^ mice.

**Figure S5. Lgmn^+^ macrophages derived EVs induces CD8^+^ T cell exhaustion through glycolytic suppression.**

(**A**) *Lgmn* mRNA levels in CD8^+^ T cells conditioned by Lgmn^fl/fl^-TAMs and Lgmn^cko^-TAMs. (**B**) Transmission electron microscopy of Lgmn^fl/fl^/EVs (left) and Lgmn^cko^/EVs (right). (**C**) Triton X and proteinase K (PK) were employed to determine the topology of Lgmn. PK treatment alone had no impact on Lgmn expression, suggesting protection by the cell membrane. However, when combined with Triton X for membrane permeabilization, Lgmn was completely degraded. (**D**) Western blot analysis of Lgmn in CD8^+^ T cells treated with Lgmn^fl/fl^/EVs or Lgmn^cko^/EVs. (**E**) Flow cytometry analysis of TIM-3^+^ and PD-1^+^ proportions in CD8^+^ T cells treated with Lgmn^fl/fl^/EVs or Lgmn^cko^/EVs. (**F**) Heatmap of differentially expressed proteins in CD8^+^ T cells treated with Lgmn^fl/fl^/EVs and Lgmn^cko^/EVs.

**Figure S6. Lgmn recruits Chip to ubiquitinate Eno1 to regulate the dysfunction of CD8^+^ T cells.**

(**A**) Co-IP assays by co-transfecting HEK-293T cells with Flag-tagged Lgmn and HA-tagged Eno1. (**B**) Western blot analysis of chip expression in CD8^+^ T cells overexpressing Lgmn. (**C**) Chip was knocked down and Lgmn was overexpressed, and lactate production in CD8^+^ T cells was detected.

**Figure S7. Safety analysis of the combined RR-11a and anti-PD-1 treatment.**

(**A**) Body weight of normal mice treated with control or the combination of RR-11a and anti-PD-1, respectively (n=5/group). (**B**) Serum biochemical indexes of normal mice treated with control or the combination of RR-11a and anti-PD-1, respectively (n=5/group). (**C**) HE staining of major organs from normal mice treated with control or the combination of RR-11a and anti-PD-1, respectively (n=5/group).

**Supplementary Table 1. Overall characteristics of enrolled NSCLC patients.**

| **Characteristic** | **Cases (n=33)** | **Responders**  **(n = 20)** | **Non-responders**  **(n =13)** |
| --- | --- | --- | --- |
| **Age (y)** |  |  |  |
| <60 | 15 (45%) | 8 (40%) | 7 (54%) |
| ≥60 | 18 (55%) | 12 (60%) | 6 (46%) |
| **Sex** |  |  |  |
| Male | 19 (58%) | 12 (60%) | 7 (54%) |
| Female | 14 (42%) | 8 (40%) | 6 (46%) |
| **Mutation status** |  |  |  |
| EGFR 19del | 3 (9%) | 1 (5%) | 2 (15.5%) |
| EGFR L858R | 3 (9%) | 1 (5%) | 2 (15.5%) |
| negative driver gene | 27 (82%) | 18 (90%) | 9 (69%) |
| **Stage** |  |  |  |
| III | 6 (18%) | 3 (15%) | 3 (23%) |
| IV | 27 (82%) | 17 (85%) | 10 (77%) |
| **Therapy** |  |  |  |
| Chemotherapy + ICI | 33(100%) | 20(100%) | 13(100%) |
